# All-Cause Mortality in Tianjin, China, 1999-2004

**Published:** 2009-09-15

**Authors:** Xiexiu Wang, Guohong Jiang, Dezheng Wang, Yi Pan, Matthew Boulton

**Affiliations:** Tianjin Centers for Disease Control and Prevention; Tianjin Centers for Disease Control and Prevention and Tianjin Medical University, Tianjin, China; Tianjin Centers for Disease Control and Prevention, Tianjin, China; Tianjin Centers for Disease Control and Prevention, Tianjin, China; University of Michigan, Ann Arbor, Michigan

## Abstract

**Introduction:**

We analyzed trends of major causes of death in Tianjin, China, from 1999 through 2004 to better inform disease prevention and control programs and policies.

**Methods:**

To report all-cause deaths among Tianjin residents from 1999 through 2004, we standardized mortality rates to the world population in 2000. We analyzed age, sex, and geographic distribution of deaths from different causes and the leading causes of death in Tianjin.

**Results:**

The 5 leading causes of death in Tianjin were cardiovascular disease, cerebrovascular disease, malignant neoplasm, chronic lower respiratory disease, and injuries and poisoning. Mortality in Tianjin declined from 0.60% in 1999 to 0.48% in 2004. Noncommunicable diseases accounted for more than 80% of all deaths. Infant and maternal mortality in Tianjin were low. Life expectancy of Tianjin residents increased every year but was consistently longer in women. When deaths from the main chronic diseases are not considered, life expectancy lengthens substantially.

**Conclusion:**

Chronic diseases are the leading cause of death in Tianjin, China. China should commit additional resources to supporting chronic disease prevention and control programs, including proven special health promotion projects.

## Introduction

Changes in the rate and causes of death are indicators of population health status and reveal changes in the spectrum of diseases that affect a population. Measuring these changes provides a scientific basis for establishing health policies and understanding health changes that occur in population groups (1). Collecting and analyzing all-cause mortality data over time help establish appropriate disease control plans, rationally allot health resources, and promote sustained social and economic development. The goal of this study was to describe patterns of all-cause mortality among residents of Tianjin, China.

## Methods

Tianjin is the third largest city in China, and its population (10 million) is approximately 40% urban and 60% rural. In 1984, the Tianjin Centers for Disease Control and Prevention (TJCDC) established an all-cause mortality reporting system for the city. We assessed 342,988 death reports of Tianjin residents collected from January 1, 1999, through December 31, 2004. Cause of death was classified according to the International Classification of Diseases, Ninth Revision (ICD-9), for 1999 through 2002 and the 10th revision (ICD-10) for 2003 and 2004. We analyzed the change and distribution of mortality, maternal mortality, infant mortality, life expectancy, and years of potential life lost.

Records were included of all people who died from 1999 through 2004 if they had formal residential household registration in Tianjin and a completed death certificate. Practicing clinicians in hospitals and community medical centers in Tianjin complete death certificates and enter the data into a computer linked with the local disease prevention station and the TJCDC. Deaths outside a hospital are added to the database on the basis of home surveys conducted by community doctors. TJCDC screens, sorts, and analyzes submitted data. TJCDC holds periodic training to improve the quality of death certificate reporting.

We calculated mortality rates according to ICD-9 or ICD-10 classification; age-standardized mortality rates are based on the World Health Organization world standard population in 2000 (2). We used χ^2^ tests for significance (α = .05) to compare the change and difference between the mortality trend and mortality distribution, respectively (3). All analyses were done in SPSS version 11.5 (SPSS, Inc, Chicago, Illinois).

## Results

We recorded 54,494, 59,575, 56,111, 56,659, 59,068, and 57,081 deaths in Tianjin for each year of the survey (1999-2004), respectively, for a total of 342,988 deaths for the 6-year period. From 1999 through 2004, all-cause mortality declined for both men (from 686.6 to 551.2 per 100,000) and women (from 509.1 to 407.2 per 100,000) (*P* < .001), although mortality was consistently higher among men than among women (*P* < .001). All-cause mortality also decreased in both urban and rural areas, but the decline was steeper in urban areas (Table 1).

Deaths from noncommunicable diseases were far more common in Tianjin during the study period than were deaths from infectious and parasitic diseases. The 5 leading causes of death were all noncommunicable diseases (cerebrovascular disease, cardiovascular disease, malignant neoplasm, chronic lower respiratory disease, and injuries and poisoning), which collectively accounted for more than 80% of all deaths (Table 2). Standardized mortality in Tianjin for cerebrovascular disease, cardiovascular disease, and malignant neoplasm all decreased from 1999 through 2004 (*P* < .001), but mortality for diabetes increased (*P* < .001).

The 3 leading causes of maternal death in Tianjin were postpartum hemorrhage (30%), preeclampsia (16%), and puerperal infections (16%). Maternal mortality differed significantly between urban and rural areas (Table 3). Infant mortality decreased from 0.8% to 0.4% during the study (*P* < .001) and, except for 2003 and 2004, was consistently lower in urban than in rural areas (Table 4). The 3 leading causes of infant death were prematurity (31%), newborn birth injuries and asphyxia (24%), and congenital heart disease (12%).

Deaths from injuries and poisoning were twice as common among men as among women in Tianjin. The leading causes of injury were traffic accidents (50%), suicide (14%), accidental poisoning (12%), drowning (7%), and falls (4%). Life expectancy of women exceeded that of men for all years of the study. Women's life expectancy increased from 79 to 82 years (0.78% per year), and men's life expectancy increased from 75 to 78 years (0.77% per year). The years of potential life lost from the most common chronic diseases increased from 592,641 to 654,408 during the study.

## Discussion

As cultures industrialize, access to treatment for infectious diseases increases, dietary and exercise patterns worsen, and the population ages, all of which shift the most common causes of death from infectious diseases to chronic diseases (4). We found that noncommunicable diseases have begun to dominate causes of death in Tianjin; the 5 leading causes of death are all chronic and account for more than 80% of all deaths (5).

Although life expectancy increased by 3 years in both men and women from 1999 through 2004, it would have increased by an additional 11 years if no one died from chronic diseases (cerebrovascular disease, cardiovascular disease, malignant neoplasm, and diabetes). Years of potential life lost from chronic diseases increased every year from 1999 through 2004. Because much chronic disease is attributed to lifestyle factors (diet, exercise, and smoking), many of these deaths could have been prevented. Unfortunately, as economic conditions improve, diets become less healthy, sedentary lifestyles become more common, and smoking becomes more prevalent (6). Among Tianjin residents aged 18 years or older, approximately 37% have hypertension and hyperlipidemia and 30% are obese (7). Injuries and poisoning are the most common causes of death among people aged 45 or younger, although years of potential life lost from injuries and poisoning are difficult to estimate (8,9).

The leading causes of death in China as a whole are similar to those in Tianjin (10,11). China's government has increased funding for disease prevention in the aftermath of the severe acute respiratory syndrome outbreak in 2003, but most of it has gone toward preventing infectious diseases. China must dedicate more resources to support health promotion and chronic disease control. A pilot program called Maintaining Normal Blood Pressure and Body Weight has been developed in urban communities to help control chronic disease. Funding, however, is inadequate for the program to benefit the large population of Tianjin, and because our findings show higher mortality from chronic disease in rural than in urban areas, health education and promotion programs must be developed for rural areas to decrease the disparity.

Our study is limited in that mortality was derived primarily from deaths reported by hospitals, and mortality is approximately 10% underreported outside the hospital system (unpublished data). Nevertheless, this study presents the first data from a provincial surveillance system on all-cause mortality in China. It provides basic information for developing health policy and health promotion activities that can improve the health and life expectancy of the Chinese people.

## Figures and Tables

**Table 1:** Mortality (per 100,000 Population) Standardized to the World Standard Population, by Sex and Urban vs Rural Residence, Tianjin, China, 1999-2004^a^

Year	Population	Urban			Rural			Total		

		Men	Women	Total	Men	Women	Total	Men	Women	Total
1999	9,101,712	685.6	523.9	604.7	686.5	487.7	587.1	686.6	509.1	597.8
2000	9,120,007	669.7	512.8	591.3	681.5	507.7	594.6	676.0	511.4	593.7
2001	9,139,761	638.6	487.4	563.0	619.5	476.8	548.2	631.2	484.2	557.7
2002	9,190,530	589.4	445.2	517.3	600.8	450.6	525.7	595.1	449.0	522.1
2003	9,321,629	590.3	447.9	519.1	586.0	439.6	512.8	589.1	445.7	517.4
2004	9,325,493	525.0	395.1	460.1	583.1	422.5	502.8	551.2	407.2	479.2

a All differences (men vs women, urban vs rural, changes over time) significant at *P* < .001 (χ^2^ test).

**Table 2:** Ten Leading Causes of Death, Tianjin, China, 1999-2004

Rank	Cause of Death (%)^a^						

	1999	2000	2001	2002	2003	2004	Total
1	CbVD (26)	CbVD (26)	CbVD (26)	CVD (24)	CVD (25)	CVD (25)	CbVD (25)
2	CVD (25)	CVD (24)	CVD (24)	CbVD (23)	CbVD (23)	CbVD (24)	CVD (24)
3	Neoplasm (19)	Neoplasm (19)	Neoplasm (20)	Neoplasm (21)	Neoplasm (20)	Neoplasm (21)	Neoplasm (20)
4	CLRD (8)	CLRD (10)	CLRD (10)	CLRD (10)	CLRD (9)	CLRD (9)	CLRD (9)
5	I&P (5)	I&P (5)	I&P (4)	I&P (4)	I&P (5)	I&P (5)	I&P (5)
6	GI disease (2)	Diabetes (2)	Diabetes (3)	Diabetes (3)	GI disease (3)	Diabetes (3)	Diabetes (3)
7	Diabetes (2)	GI disease (2)	GI disease (3)	GI disease (3)	Diabetes (3)	GI disease (3)	GI disease (3)
8	GU disease (2)	GU disease (2)	GU disease (2)	GU disease (2)	GU disease (2)	GU disease (2)	GU disease (2)
9	CNS disease (1)	CNS disease (1)	CNS disease (1)	CNS disease (1)	CNS disease (1)	CNS disease (1)	CNS disease (1)
10	Perinatal death (<1)	Perinatal death (<1)	Infection (<1)	Infection (<1)	Hereditary malformation (<1)	Perinatal death (<1)	Infection (<1)

Abbreviations: CbVD, cerebrovascular disease; CVD, cardiovascular disease; CLRD, chronic lower respiratory disease; I&P, injuries and poisoning; GI, gastrointestinal; GU, genitourinary; CNS, central nervous system.

a Percentages may not total 100 because of rounding.

**Table 3:** Trends in Maternal Mortality, by Urban vs Rural Residence, Tianjin, China, 1999-2004

Year	Maternal Mortality (per 100,000 Population)^a^		

	Urban	Rural	Total
1999	7.5	14.7	11.5
2000	10.5	2.7	6.1
2001	18.9	7.3	11.9
2002	6.8	12.6	10.4
2003	5.2	13.4	10.6
2004	15.6	12.0	13.2

a χ^2^ tests between urban and rural residence, *P* = .04.

**Table 4:** Trends in Infant Mortality, by Urban vs Rural Residence, Tianjin, China, 1999-2004

Year	Infant Mortality (per 1,000 Births)^a^		

	Urban	Rural	Total
1999	6.5	8.6	7.7
2000	4.3	5.9	5.2
2001	4.6	5.5	5.1
2002	4.1	4.4	4.3
2003	4.9	4.7	4.8
2004	4.9	4.2	4.4

a χ^2^ test for annual trend, *P* < .001; between urban and rural residence, *P* = .03.
